# Uncovering the Potential of Termite Gut Microbiome for Lignocellulose Bioconversion in Anaerobic Batch Bioreactors

**DOI:** 10.3389/fmicb.2017.02623

**Published:** 2017-12-22

**Authors:** Lucas Auer, Adèle Lazuka, David Sillam-Dussès, Edouard Miambi, Michael O'Donohue, Guillermina Hernandez-Raquet

**Affiliations:** ^1^Laboratoire d'Ingénierie des Systèmes Biologiques et des Procédés, Université de Toulouse, Centre National de la Recherche Scientifique, Institut National de la Recherche Agronomique, INSA, Toulouse, France; ^2^Laboratoire d'Éthologie Expérimentale et Comparée, Université Paris 13 - Sorbonne Paris Cité, Villetaneuse, France; ^3^Institut d'Ecologie et des Sciences de l'Environnement de Paris, Institut de Recherche Pour le Développement – Sorbonne Universités, Bondy, France; ^4^Institut d'Ecologie et des Sciences de l'Environnement de Paris, Université Paris-Est Créteil, Créteil, France

**Keywords:** termite, gut-microbiome, lignocellulose bioconversion, carboxylate production, microbial diversity

## Abstract

Termites are xylophages, being able to digest a wide variety of lignocellulosic biomass including wood with high lignin content. This ability to feed on recalcitrant plant material is the result of complex symbiotic relationships, which involve termite-specific gut microbiomes. Therefore, these represent a potential source of microorganisms for the bioconversion of lignocellulose in bioprocesses targeting the production of carboxylates. In this study, gut microbiomes of four termite species were studied for their capacity to degrade wheat straw and produce carboxylates in controlled bioreactors. All of the gut microbiomes successfully degraded lignocellulose and up to 45% w/w of wheat straw degradation was observed, with the *Nasutitermes ephratae* gut-microbiome displaying the highest levels of wheat straw degradation, carboxylate production and enzymatic activity. Comparing the 16S rRNA gene diversity of the initial gut inocula to the bacterial communities in lignocellulose degradation bioreactors revealed important changes in community diversity. In particular, taxa such as Spirochaetes and Fibrobacteres that were highly abundant in the initial gut inocula were replaced by Firmicutes and Proteobacteria at the end of incubation in wheat straw bioreactors. Overall, this study demonstrates that termite-gut microbiomes constitute a reservoir of lignocellulose-degrading bacteria that can be harnessed in artificial conditions for biomass conversion processes that lead to the production of useful molecules.

## Introduction

With more than 200 billion tons of non-food lignocellulosic biomass produced yearly, lignocellulose represents the most abundant source of renewable carbon on Earth (Chandel and Singh, 2011). For this reason, the bioconversion of lignocellulose into biofuels or industrial chemicals is receiving much attention, because this is one route toward creating a fossil carbon-free economy.

Lignocellulose is a composite material, composed of cellulose microfibrils, hemicellulose, lignin and a variety of other minor components (Lynd et al., 2002). Each of these polymers displays intrinsic complexity and together they form a highly recalcitrant material. In natural ecosystems, the degradation of lignocellulose involves chemical processes and a large arsenal of enzymes, including cellulases, hemicellulases, and lignin-degrading enzymes (Cragg et al., 2015). To develop industrial lignocellulose bioconversion processes, it is necessary to overcome the recalcitrant nature of lignocellulosic biomass, while limiting cost as much as possible. So far, this trade-off has not been satisfied and current processes are not cost competitive at industrial scale.

The degradation and recycling of lignocellulose in natural ecosystems is performed by various organisms, including bacteria, fungi and animals (Cragg et al., 2015). Therefore, the in-depth study of these deconstruction strategies is a useful way to identify and understand biomass bioconversion processes. Most plant biomass degradation occurs in the soil, where it is partially mineralized, producing CO_2_ and humic substances (Deacon, 2005). The principal players in soil-based biomass degradation are aerobic fungi and bacteria that produce extracellular biomass-degrading enzymes (Fierer et al., 2009; Burns et al., 2013). Owing to the tremendous ability of filamentous fungi to secrete large quantities of biomass-hydrolyzing enzymes, fungi are currently the main sources of industrial hydrolytic enzymes used for biorefinery purposes (Kubicek and Kubicek, 2016). Regarding animals, many herbivores and omnivores are actually devoid of endogenous lignocellulolytic enzymes and rely upon microbial symbionts to supply the requisite enzymatic arsenal (Smant et al., 1998). One of the best examples of this symbiosis is that of ruminant animals where bacteria, archaea, protozoa, and anaerobic fungi act in concert to degrade lignocellulose and ultimately produce short chain carboxylates (volatile fatty acids, VFA) and methane (Hobson, 1998). Since VFA and methane are commercially valuable molecules the harnessing of rumen fermentation for biotechnological processes has already been assessed using, for example, a rumen inoculum to enhance methane production in bioreactors using various lignocellulosic feedstocks (Yue et al., 2007).

Wood-eating termites figure among the animals that are best equipped for the digestion of lignocellulosic material. Although the feeding regimes of different termite species vary, some species are able to digest crystalline cellulose and overcome the lignin barrier (Breznak and Brune, 1994). Moreover, compared to ruminants, termites are able to degrade wood more efficiently, since they remove 74–99% cellulose and 65–87% hemicellulose from wood samples (Brune, 2014). For this reason, it is thus unsurprising that termites are considered as promising sources of plant cell wall-degrading microorganisms and enzymes.

It has long been acknowledged that termites belong to the order Isoptera, but more recent work suggests that they are related to cockroaches and are thus members of the Blattodea order (Inward et al., 2007; Lo et al., 2007). Termites have been classified into two major groups, lower (families Archotermopsidae, Masto-, Stolo-, Kalo-, Hodo-, Stylo-, Rhino- and Serri-termitidae) and higher (family Termitidae) termites (Inward et al., 2007). The members of these groups display different lignocellulose digestion strategies. Lower termites rely on complex symbiotic interactions with eukaryotic flagellates and bacteria (Ni and Tokuda, 2013), whereas higher termites rely on an external symbiotic interaction with fungal species of *Basidiomycetes* (e.g., termites from the subfamily *Macrotermitinae*) and/or harbor a mutualistic hindgut microbiome exclusively constituted by prokaryotes (Slaytor, 1992; Tokuda and Watanabe, 2007; Brune, 2014). Additionally, several lower and higher termite species appear to produce endogenous cellulases, notably in the buccal cavity (Watanabe et al., 1998; Lo et al., 2010). Unlike rumen and cellulolytic soil bacterial communities, which are dominated by Firmicutes and Bacteroidetes, the particle-associated bacteria in wood-feeding termite guts are mainly constituted by Spirochaetes and Fibrobacteres (Mikaelyan et al., 2014). This marked difference probably reflecting distinct degradation mechanisms and bioconversion pathways.

Driven by technological goals, interest in termites has increased in recent years, extending investigations beyond the entomological community. Many studies have focused on the identification and characterization of enzymatic arsenals from different termite species (König et al., 2013), with some using advanced metagenomics to explore termite gut microbiomes from higher termites that feed on plant biomass at different stages of decomposition (Warnecke et al., 2007; Bastien et al., 2013; Rossmassler et al., 2015; Scharf, 2015). However, fewer studies have actually focused on harnessing termite gut microbiomes, using them as inocula for biotechnological applications (Hamdi et al., 1992), despite their natural ability to produce industrially relevant carboxylates (Agler et al., 2011).

The carboxylate platform is an interesting alternative to the more common biomass to ethanol route and could be useful to diversify lignocellulose biorefining (Holtzapple et al., 1999; Agler et al., 2011). In the carboxylate platform, anaerobic mixed bacterial communities growing under non-sterile conditions are used to produce acetic, propionic and butyric acid, all of which are interesting platform chemicals for further biological or chemical transformations (Kleerebezem and van Loosdrecht, 2007; Agler et al., 2011). Nevertheless, to develop a feasible carboxylate platform technology, it is first necessary to stabilize a suitable lignocellulose-degrading microbial community in artificial bioreactor conditions. Numerous attempts to achieve this have used lignocellulolytic communities from soil (Feng et al., 2011; DeAngelis et al., 2012), compost (Guo et al., 2010; Reddy et al., 2013), marine sediments (Hollister et al., 2011), or extreme environments (Cope et al., 2014). However, so far the lignocellulose conversion yields obtained have been insufficient to envisage commercial viability. Moreover, the fundamental knowledge concerning the microbiological processes that underpin carboxylate production on lignocellulosic biomass remains insufficient to make further progress. Therefore, to extend the knowledge base, the present study assessed the use of gut microbiomes from higher termites for the production of carboxylates in controlled bioreactors. Gut microbiomes from four termite species were evaluated for their ability to produce carboxylates from wheat straw in controlled bioreactors, monitoring substrate composition, products, enzymatic activities and microbial community dynamics using 16S rRNA gene sequencing.

## Materials and methods

### Lignocellulose substrate and termite gut inocula

Wheat straw from the winter wheat variety Koreli was collected at an experimental farm (INRA, Boissy-le-Repos, France) in August 2011. After harvesting, the straw was milled to 2 mm and stored at room temperature (20–25°C).

Four different species of higher termites (Termitidae family) *Microcerotermes parvus, Termes hospes, Nasutitermes ephratae*, and one undescribed species closely related to *N. lujae* (herein after *N. lujae*) were selected as inocula for this study. The major selection criteria were based on the fact that higher termites harbor a predominantly bacterial gut microbiome and that the intestinal pH in each species is slightly acidic or near to neutrality (Köhler et al., 2012). *M. parvus, N. ephratae*, and *N. lujae* are wood-feeding termites while *T. hospes* is a soil-feeding species (Eggleton et al., 1995). Other selection criteria were prior knowledge on their lignocellulose degradation ability, acetate production, and the availability of the species at the Institut de Recherche pour le Developement (IRD, Bondy, France).

Termite colonies were maintained in a temperature-controlled room (27°C, 90% relative humidity) at IRD. One thousand worker termites per species were randomly collected from their nest in two campaigns separated by a period of 3 months (i.e., sample size *n* = 500 guts per species per sampling campaign). Following cold anesthesia of termites, whole guts were removed by dissection on ice with sterile scissors and forceps. Once removed, the guts were immediately suspended in a physiological saline solution (PBS-buffer) and maintained on ice (Santana et al., 2015). Once completed, gut samples (containing 500 guts each) were frozen at −20°C, transported to LISBP on dry ice and stored at −80°C. Due to the low number of termite-gut available, for DNA extraction, a small fraction of guts (20 guts) per species were collected separately (in duplicate) and stored as previously described. Similar low numbers of insect-guts have been used in previous studies (Scully et al., 2013; Marynowska et al., 2017). Finally, samples composed of 10 guts per species were weighed to estimate the biomass contained within a typical inoculum.

### Anaerobic bioreactors

To assess the lignocellulose degradation capacity of the different gut microbiomes, two replicate anaerobic bioreactors (Applikon MiniBio 500) were conducted for each termite species. Following centrifugation (7,197 × g, 10 min, 4°C) and elimination of the saline media, termite-gut homogenates (500 guts) were used to inoculate 400 mL of mineral media (MM), containing per liter of distilled water: KH_2_PO_4_, 0.45 g; K_2_HPO_4_, 0.45 g; NH_4_Cl, 0.4 g; NaCl, 0.9 g; MgCl_2_.6H_2_O, 0.15 g; CaCl_2_.2H_2_O, 0.09 g, supplemented with 250 μL of V7 vitamin solution (Pfennig and Trüper, 1992) and 1 mL of sterilized (0.2 μm filtration) trace elements solution (containing per liter of distilled water: H_3_BO_3_, 300 mg; FeSO_4_.7H_2_O, 1.1 g; CoCl_2_.6H_2_O, 190 mg; MnCl_2_.4H_2_O, 50 mg; ZnCl_2_, 42 mg; NiCl_2_.6H_2_O, 24 mg; NaMoO_4_.2H_2_O, 18 mg; CuCl_2_.2H_2_O, 2 mg). Milled wheat straw (2 mm) was sterilized by autoclaving (120°C, 20 min and 1.2 bars) and added to the medium (20 g.L^−1^) as the sole carbon source. Stirred bioreactors (400 rpm) were operated under strict anaerobic conditions, flushing the reactor with nitrogen after inoculation. The absence of dissolved oxygen was continuously monitored with a polarographic dissolved oxygen probe (AppliSens). The temperature was set to 35°C and pH was maintained at 6.15 by adding a 2 M NaOH solution. During the incubation, methane was monitored and, if necessary, inhibited by addition a solution of 2*-*bromoethanesulfonate (BES), a methanogenesis inhibitor, until a maximum concentration of 10 mM. A non-inoculated control bioreactor was operated under identical conditions. Samples were collected from all bioreactors every 2 days during the 20 days of incubation to characterize VFA and gas production. Samples from the initial and final times of incubation were used to determine LC degradation, enzymatic activities and microbial diversity.

### Chemical analysis

Gas production was measured by monitoring pressure changes and gas composition was analyzed using a gas chromatograph (GC) HP 5890 equipped with a conductivity detector and a HAYSEP D column (molecular sieve of 5 Å). Argon was used as the carrier gas at a flow rate of 100 mL.min^−1^. Injector, oven, and detector temperatures were 100, 60, and 140°C, respectively.

Volatile Fatty Acids (VFA) contained in the liquid phase of samples were analyzed using a Varian 3900 gas chromatograph equipped with a flame ionization detector and CP-Wax 58 (FFAP) CB column (length: 25 m, inside diameter: 0.53 mm).

Wheat straw concentration was determined at the beginning and at the end of the 20-day incubation by measuring the total (TS) and volatile (VS) solids. TS were determined using 10 mL samples that were first centrifuged (7,197 × g, 10 min), rinsed twice with distilled water and dried for 24 h at 105°C. The mineral fraction (MF) was estimated by mineralization of the samples at 500°C for 2 h, and VS were determined by subtracting MF from TS. Wheat straw degradation was reported as percentage of LC (%, w/w) related to the initial LC mass.

### Enzyme activity assay

Enzyme activity measurements were performed as previously described by Lazuka et al. (2015). Briefly, triplicate bioreactor samples (5 mL) were removed at the end of the experiment (after 20 days) and centrifuged at 7,197 × g for 10 min at 4°C. Enzyme activity detected within the supernatant was designated as “extracellular enzymes,” while enzyme associated with the pellet was designated “cell-bound enzymes.” Xylanase and endoglucanase (CMCase) activities were measured using 1% w/v xylan beechwood (Sigma) and 1% w/v carboxymethyl cellulose (CMC purchased from Sigma, France), respectively. Activities were estimated by the DNS method. One unit of xylanase or CMCase activity (UA, unit of activity) was defined as the amount of enzyme that produces 1 μmol of reducing sugars per minute in the measurement conditions. These measurements were only possible for the second replicate bioreactors.

### 16S rRNA gene copy number and diversity analysis

16S rRNA gene copy number and bacterial diversity were analyzed on the initial termite gut samples and at the end of the bioreactor experiments. Samples (1.5 mL) were collected and centrifuged at 13,000 × g, for 5 min at 4°C. After removing the supernatant, the pellet was snap frozen in liquid nitrogen and stored at −80°C. Total DNA/RNA was extracted from these samples using a PowerMicrobiome RNA Isolation kit (MoBio Laboratories Inc. Carlsbad) following the manufacturer's instructions, but omitting the final DNAse steps. Cell lysis was carried out using a Fast Prep (MP Biomedicals) (2 × 30 s at 4 ms^−1^). DNA was purified using an AllPrep DNA/RNA Mini Kit (Qiagen) according to the manufacturer's instructions. DNA integrity and purity were checked using agarose gel (1%) electrophoresis. DNA concentration was measured using a NanoDrop 1000 spectrophotometer (Thermo Scientific), measuring absorbance at 260 and 280 nm.

16S rRNA copy number was determined by qPCR using a Realplex Mastercycler (Eppendorf, Montesson, France). For each sample, assays were carried out in triplicate using 96-well real-time PCR plates (Eppendorf). The qPCR was performed in 25 μL containing 12.5 μL Master Mix (Invitrogen, Eugen, USA), using primers BAC3388 and BAC805R (250 nM of each primer), the TaqMan probe BAC516F (100 nM) and DNA template ranging from 10–100 ng as previously described (Yu et al., 2005). Real-time PCR thermocycling was conducted at 95°C for 20 s during 1 cycle, then at 95°C for 15 s during 40 cycles and finally at 60°C for 1 min. A negative control without DNA template was subjected to the same procedure in order to monitor contamination. A standard curve was generated for each assay using 10-fold dilutions of pEX-A plasmid (Eurofins MWG Operon) containing the target gene sequence. Three different dilutions of each sample were amplified and the initial concentrations were calculated from reactions displaying satisfactory PCR amplification.

Microbial diversity was analyzed using MiSeq Illumina sequencing, performed at the GenoToul Genomics and Transcriptomics facility (GeT, Auzeville, France). The V3-V4 hypervariable region of the 16S rRNA gene was amplified from genomic DNA samples using the bacterial primers 343F (5′-CTT TCC CTA CAC GAC GCT CTT CCG ATC TAC GGR AGG CAG CAG-3′) and 784R (5′-GGA GTT CAG ACG TGT GCT CTT CCG ATC TTA CCA GGG TAT CTA ATC CT-3′) modified to add adaptors during the second PCR amplification. The first PCR amplification was performed in 50 μl reactions containing 1X PCR buffer, 2.5 U MTP Taq DNA Polymerase (Sigma), 0.2 mM of each dNTP, 0.5 mM of each primer and 2 ng of extracted DNA. After 30 amplification cycles (94°C, 1 min; 65°C, 1 min; 70°C, 1 min), amplicons were purified using magnetic beads and quantified using a NanoDrop 1000 spectrophotometer. A second PCR amplification was performed at the GeT platform to add sequencing adapters and a unique index for each sample (details in Supplementary Data). The PCR products were purified using magnetic beads and their quality was ascertained using a High Sensivity DNA Analysis Kit (Agilent) and a BioAnalyzer 2100. DNA concentration was measured using a NanoDrop 1000 spectrophotometer. An equimolar pool was then prepared and loaded on a MiSeq Illumina cartridge, using reagent kit v3 which enabled paired 300-bp reads.

Sequencing data were demultiplexed at the GeT platform and pair-ends reads were joined with Flash v1.2.6 (Magoč Salzberg and Salzberg, 2011), using an overlap (>110 bp) displaying a maximum ratio of 0.1 mismatches, which generated high quality full-length reads of the V3 and V4 regions. All fastq files were then merged into a unique fasta file, and processed using the software package Mothur v1.33.1 following the standard procedures (Schloss et al., 2009; Auer et al., 2017). Briefly, sequences presenting a primer mismatch or displaying unexpected length (>380 or <460 bp) were removed. To reduce the computational costs, sequences were de-replicated and unique sequences were aligned with the SILVA database (Yilmaz et al., 2014). Only the sequences that aligned with the expected V3-V4 region were further analyzed. Sequences that presented <5 differences with a more abundant one were considered as sequencing errors and were merged together. Chimeras were detected and removed using Uchime (Edgar et al., 2011) set to self-reference and default parameters for each of the sample groups. Sequences were then clustered at 3% distance which approximately corresponds to the species level (Stackebrandt and Goebel, 1994). Taxonomic affiliations were obtained using the Wang method and a fusion of LTP v115 (Yarza et al., 2008) and DictDB (Mikaelyan et al., 2015b) databases. According with Bokulich et al. (2013), in order to avoid false and/or contaminant OTUs, rare OTUs containing <20 sequences across all samples (representing <0.005% of total sequences) were removed, considering this value as a detection threshold. All OTUs validating this criterium were thus considered as true OTUs. All samples were normalized by random subsampling to a level which enable to cover the community diversity (herein 15,000 sequences per sample; see Supplementary Data S1). Abundance tables with affiliation and rarefaction curves were generated using Mothur (Schloss et al., 2009). Major OTUs are frequently defined as those displaying abundances >1%; such value, based on low resolution PCR-dependent technologies is sometimes controversial (Casamayor et al., 2000; Poretsky et al., 2014). Herein, in order to take account the eventual variability between termite-gut replicates, OTU abundance was set at >2% to define abundant OTUs.

Abundance tables, taxonomy files and phylogenetic trees were manually imported into R (v3.0.3) package Phyloseq v1.6.1 (McMurdie and Holmes, 2013). Weighted-Unifrac distances were calculated using Phyloseq and clustered using Hclust. PCoA plots were generated using Phyloseq using the vegan::metaMDS function. ClustalOWS alignment and calculation of neighbor-joining or average distance trees were performed using Jalview v2.8.2 (Waterhouse et al., 2009).

Sequence data were deposited in the sequence read archive (SRA) of the National Center for Biotechnology Information (NCBI) under accession number SRP119642">SRP119642.

## Results

### Reactor performance: wheat straw degradation and carboxylate production

The ability of gut microbiomes from four different species of higher termites (*N. ephratae, N. lujae, T. hospes* and *M. parvus*) to degrade wheat straw as sole carbon source was assessed in controlled bioreactors, performing duplicates for each termite species (named r1 and r2). The quantity of DNA in each inoculum (500 guts) was estimated to be about 150 μg, implying that each inoculum contained a similar number of 16S rRNA gene copies (~1.5 × 10^12^). However, regarding *T. hospes* these values were lower (67.9 ± 0.7 μg and 1.7 ± 0.7 × 10^11^ 16S rRNA copies; Table 1).

**Table 1:** Microbial biomass concentration estimated as 16S rRNA gene copies measured at the beginning and at the end of the incubation.

**16S rRNA gene copies/μL**				
**Species**	**initial**		**final**	
	**r1**	**r2**	**r1**	**r2**
*M. parvus*	5.0 ± 0.6 E+06	4.3 ± 0.3 E+06	5.4 ± 0.8 E+06	1.4 ± 0.3 E+07
*N. ephratae*	3.9 ± 0.2 E+06	3.7 ± 0.6 E+06	8.6 ± 1.2 E+06	2.3 ± 0.5 E+07
*N. lujae*	2.8 ± 0.4 E+06	3.9 ± 0.9 E+06	1.65 ± 0.08 E+07	1.7 ± 0.2 E+07
*T. hospes*	4.3 ± 0.7 E+05	1.4 ± 0.3 E+06	1.0 ± 0.4 E+07	7.8 ± 0.5 E+06

*Values are detailed for each inoculum and biological replicates*.

**Figure 1:**
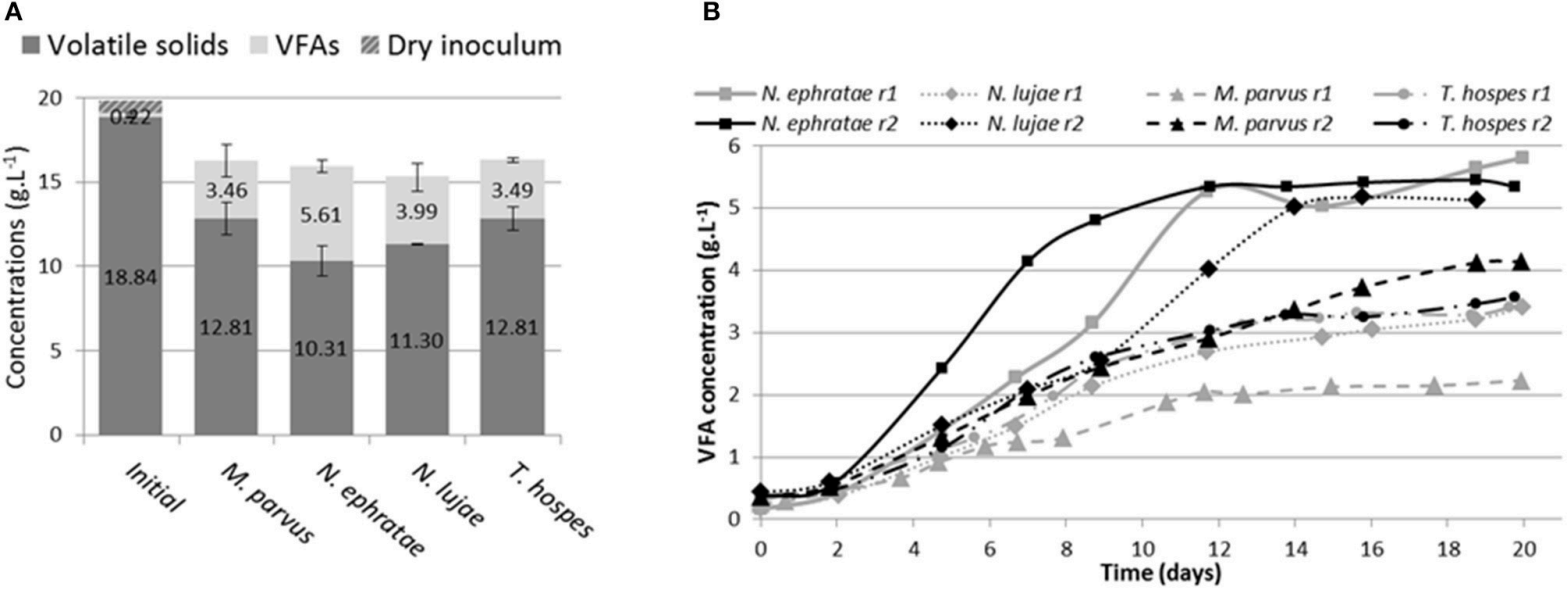
Bioreactor composition **(A)** at the beginning and at end of incubation of termite gut microbiomes issued from *Nasutitermes ephratae, N. lujae, Microcerotermes parvus* and *Termes hospes*. Errors are standard deviations of the two biological replicates. Kinetic of volatile fatty acid (VFA) production **(B)** through the incubation in each reactor (r1 and r2). Sterile wheat straw was inoculated with 500 termite guts from each species and incubated for 20 days.

After 20 days of incubation in anaerobic batch bioreactors, wheat straw degradation varied from 26 to 49% w/w for the different termite gut inocula (Figure 1A). Highest wheat straw degradation (45.2 ± 5%) was achieved by the *N. ephratae* inoculum, followed by those of *N. lujae, T. hospes*, and *M. parvus*, with mean degradation values of 37.1 ± 4.3, 30 ± 5, and 31 ± 3.7%, respectively (Figure 1A). The main products of lignocellulose bioconversion were carboxylates (VFA, Figure 1B) and CO_2_ (data not shown), irrespective of the inoculum used. Small amounts of methane were detected in some of the bioreactors, but in this case its production was immediately inhibited by the addition of BES. During the initial phase of the culture, small amounts of H_2_ production were also detected, but this ceased at later stages (data not shown). VFA accumulation varied from 2.2 to 5.8 g.L^−1^ for the different termite gut inocula, with the *N. ephratae* gut microbiome yielding the highest concentration (Figure 1B). For all the termite species tested, VFA was mainly composed of acetate (>85%), associated with smaller amounts of propionate and butyrate, the only exception being the bioreactor inoculated with *M. parvus* gut microbiome (20% propionate production). It is also noteworthy that VFA production by *N. lujae* and *M. parvus* gut inocula displayed high variability between replicates (Figure 1B) and VFA production kinetics varied between the different inocula. However, despite these differences, VFA production reached a maximum value in all bioreactors after 20 days of incubation. Importantly, the amounts of VFA produced in the different bioreactors correlated with measured lignocellulose degradation and were consistent with theoretical lignocellulose conversion yields (Lazuka et al., 2015). Logically, being devoid of inoculum the control bioreactor did not produce VFA or gas over the whole incubation period and the amount of lignocellulosic biomass remained unchanged (data not shown).

**Figure 2:**
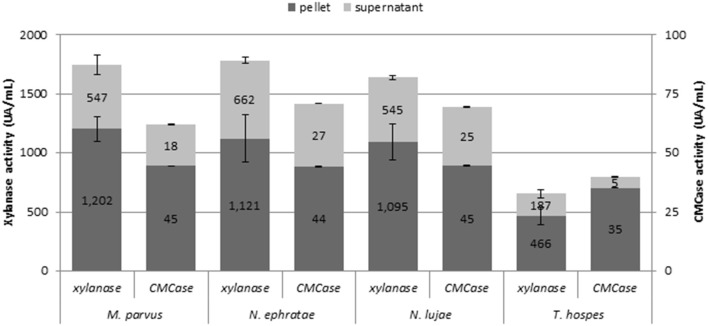
Xylanase and CMCase enzymatic activities at final the end of the incubation for the termite gut inocula from *Nasutitermes ephratae, N. lujae, Microcerotermes parvus*, and *Termes hospes*. Supernatants represent extracellular enzymatic activities whereas pellets correspond to cell-bound activities. Errors bars are standard deviations between technical replicates.

Monitoring various enzymatic activities on r2 duplicates at the end of the incubation (Figure 2) revealed free- and cell-bound xylanase and cellulase activities. However, exoglucanase and β-glucosidase activities were not detected. While bioreactors inoculated with termite guts of *N. ephratae, M. parvus* and *N. lujae* displayed significant xylanase activity (>1.500 UA.mL^−1^), the one containing the *T. hospes* inoculum displayed lower xylanase activity (600 UA.mL^−1^). Regarding CMCase activity, a similar profile was observed in all bioreactors, but the measured activity was generally lower than xylanase activity (i.e., 62–71 UA.mL^−1^ CMCase activity for *N. ephratae, M. parvus* and *N. lujae* gut inocula and 40 UA.mL^−1^ for *T. hospes* inoculum). Finally, it was noted that for all termite gut inocula, more than 60% of the xylanase and cellulose activities were cell-bound.

### Diversity analysis of the termite-gut inocula and lignocellulose degradation bioreactors

The microbial diversity of the original termite gut microbiome and that of the communities developed in the bioreactors was assessed by sequencing the V3-V4 16S rRNA gene region of genomic DNA samples. A total of 870,449 pair-end reads were successfully assembled into sequences of about 450 bp length, with an average of 54,400 reads per sample. After filtering and chimera removal, more than 20,000 high quality sequences per sample remained for further analysis. Sequence clustering yielded a total of 8,794 bacterial operational taxonomic units (OTUs) at 97% sequence similarity threshold. Rare OTUs were filtered generating 671 final OTUs. Rarefaction curves, based on normalization by subsampling at 15,000 sequences per sample, showed that all samples (except *N. lujae* r1) were close to saturation (Figure S1, Supplementary Data), indicating that all communities were sufficiently sampled to estimate bacterial diversity and richness.

### Termite gut microbiome composition

The average number of OTUs observed was 150, 181, 192, and 292 for the gut microbiomes of *T. hospes, N. ephratae, N. lujae*, and *M. parvus*, respectively (Table 2). Considering Shannon's and Simpson's reciprocal index, *T. hospes* displayed the highest diversity, while the other termite gut microbiomes displayed similar levels of diversity.

**Table 2:** Alpha-diversity indexes of gut inocula and final reactor communities.

**Species**	***M. parvus***				***N. ephratae***				***N. lujae***				***T. hospes***			
	**gut1**	**gut2**	**r1**	**r2**	**gut1**	**gut2**	**r1**	**r2**	**gut1**	**gut2**	**r1**	**r2**	**gut1**	**gut2**	**r1**	**r2**
ObsOTUs	154	147	63	52	194	169	67	58	196	186	116	44	304	281	61	63
Shannon	3.40	3.31	2.48	1.79	3.43	3.46	2.49	2.10	3.36	3.41	2.20	2.01	4.96	4.94	2.22	2.54
Simpson	12.3	10.1	7.7	3.9	13.2	12.9	7.0	5.4	13.0	12.9	4.9	5.8	64.4	65.7	5.6	7.63

Abundant OTUs were defined herein as those displaying a minimum 2% abundance (min. 300 sequences) in at least one sample. From the 34 abundant OTUs detected across all gut samples, only one was common to three termite species *N. lujae, N. ephratae*, and *M. parvus*. One OTU was common to *M. parvus* and *T. hospes*, while nine OTUs were common to the two *Nasutitermes* gut microbiomes (Supplementary Data, Table S1). Therefore, the majority of abundant OTUs were specific to a given host, and the shared OTUs were mainly associated with the two closely related *Nasutitermes* species. This observation was also true for OTUs displaying low abundances (<2%; defined as minor OTUs). Indeed, only 8 minor OTUs were common to the four termite species.

**Figure 3:**
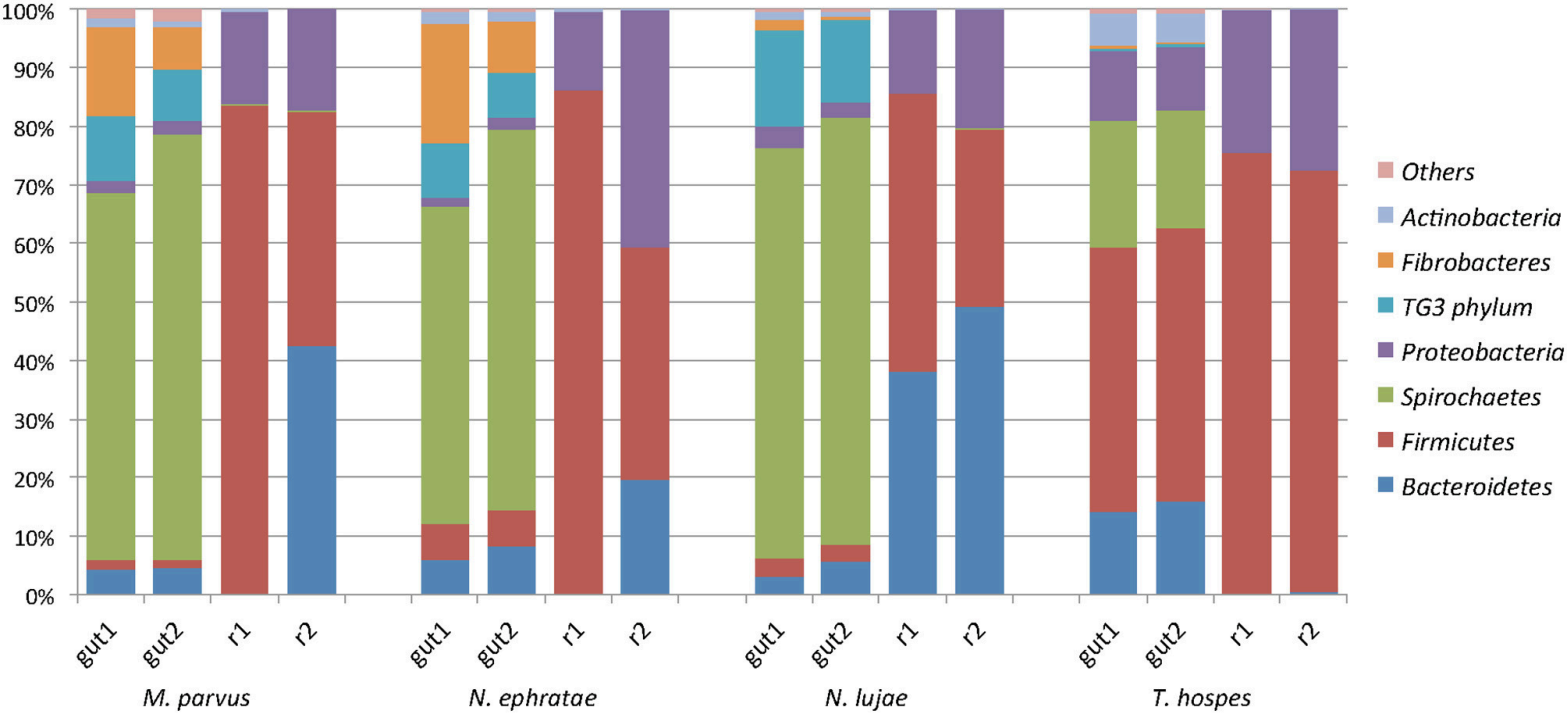
Diversity of the termite-derived microbiome. Phylum level classification of the 16S rRNA genes in the studied termite gut species and in the two replicated reactors inoculated with such guts at the end of the incubation. The category “Others” contains the low abundance phyla Acidobacteria, Chloroflexi, Cyanobacteria, Chlorobi, and Deferribacteres.

**Figure 4:**
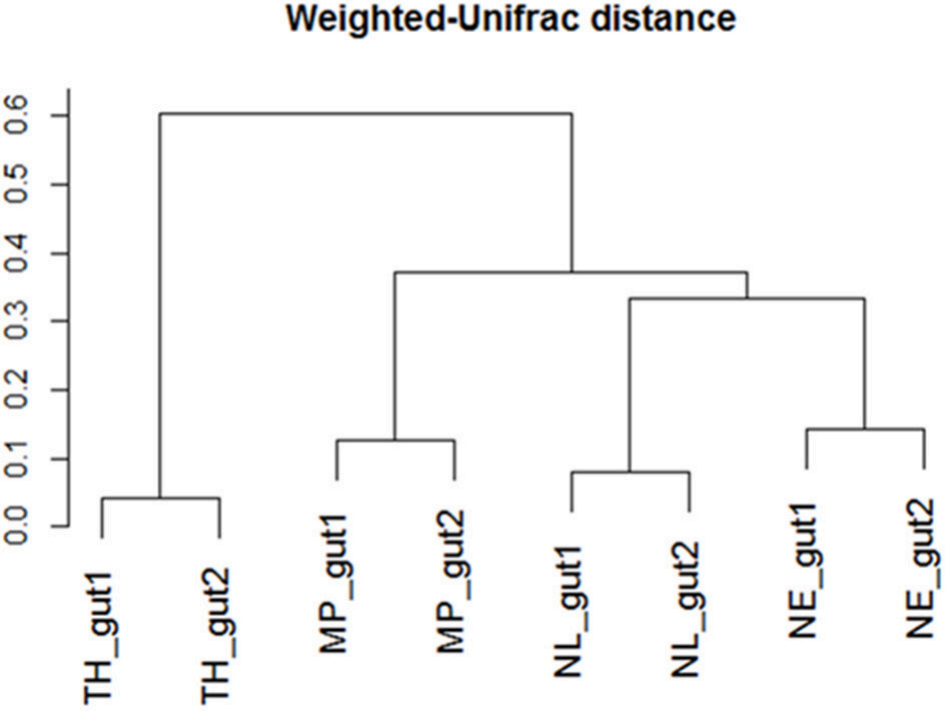
Weighted-Unifrac diversity distances between the initial termite gut communities from *Nasutitermes ephratae* (NE), *N. lujae* (NL), *Microcerotermes parvus* (MP), and *Termes hospes* (TH).

Regarding the relative abundances of species, the gut microbiome from *M. parvus* and the two species of *Nasutitermes* were dominated by Spirochaetes (>55%), followed by Fibrobacteres (except for *N. lujae*) and the candidate phylum Termite group 3 (TG3; Figure 3). A completely different profile was observed for the *T. hospes* sample, which was dominated by Firmicutes (46%), Spirochaetes (21%), Bacteroidetes (13%) and Proteobacteria (12%). Overall, these results are consistent with previous descriptions of termite gut bacterial communities (Thongaram et al., 2005; Hongoh et al., 2006; Brune, 2014; Mikaelyan et al., 2017). Weighted Unifrac distances of OTUs (Figure 4) showed that the two *Nasutitermes* gut microbiomes were closely related, while the *M. parvus* gut microbiome was closer to those of *Nasutitermes* than the gut microbial community of *T. hospes*. Based on cytochrome oxidase subunit II gene (Legendre et al., 2008), the genus *Termes* is phylogenetically closer to *Nasutitermes* than *Microcerotermes* (Supplementary Data S2). Nevertheless, the *Termes* gut microbiome was clearly different to that of *Nasutitermes* gut, being the only termite species displaying abundant OTUs belonging to Firmicutes and Proteobacteria.

### Diversity changes after incubation in lignocellulose bioreactors

Determination of observed richness (Table 2) using a 97% similarity threshold revealed that this was decreased by a factor of 2 (147–304 OTUs being reduced to 67 OTUs) over the 20-day incubation period. Moreover, for each of the termite-gut inocula, the Shannon and Simpson reciprocal diversity indices were higher than 3 and 10, respectively, whereas these values were lower than 2.6 and 7.7 for the communities sampled in the bioreactor after incubation. Taken together, these data reveal that growth of the termite gut bacterial communities in artificial bioreactor conditions using wheat straw as the sole carbon source systematically decreased microbial diversity.

**Figure 5:**
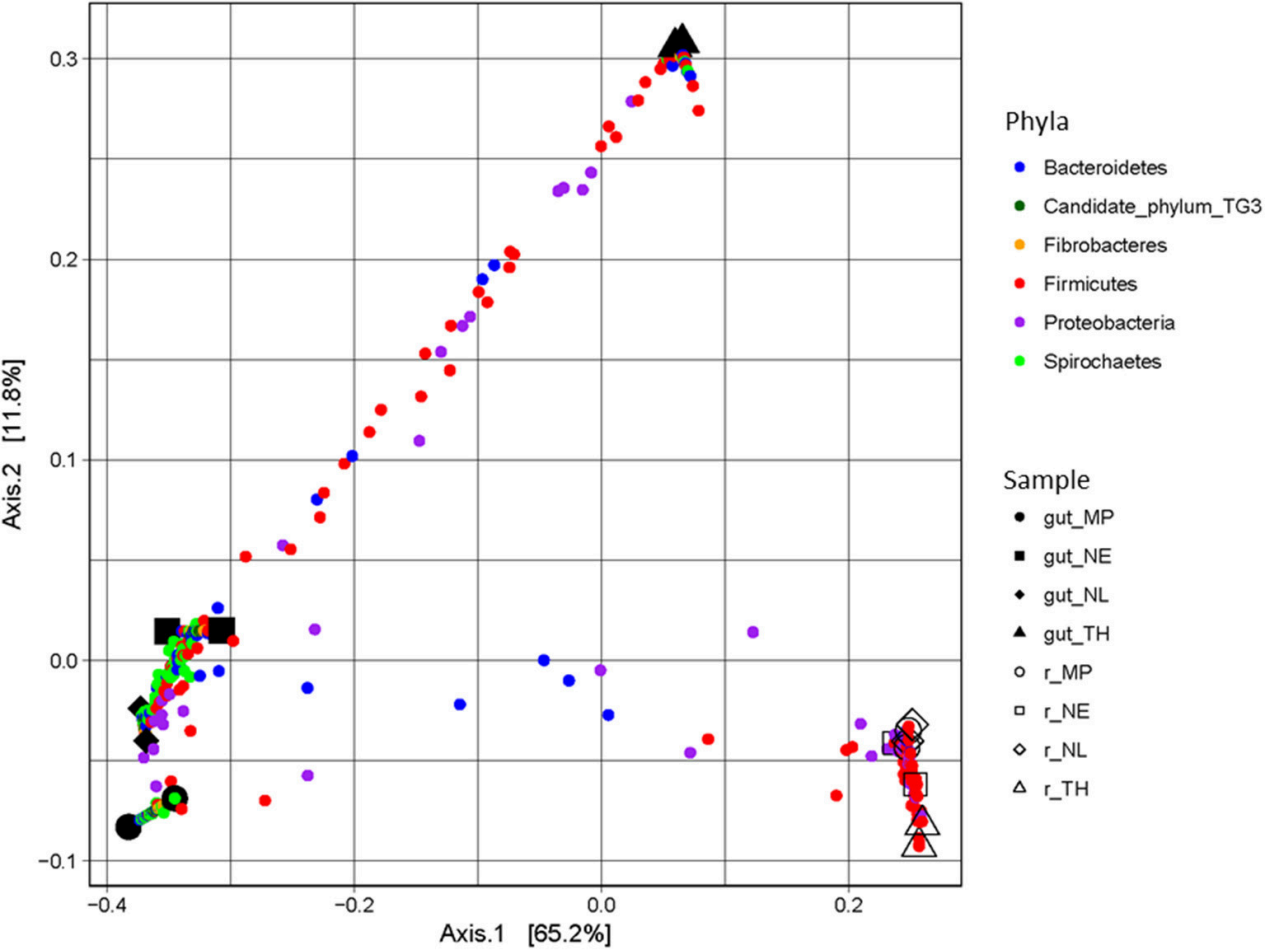
PCoA plot of weighted-Unifrac distances of guts and bioreactor communities issued from *Nasutitermes ephratae* (NE), *N. lujae* (NL), *Microcerotermes parvus* (MP), and *Termes hospes* (TH). Shapes correspond to different samples from guts and bioreactors. Small dots are colored according to their phylum, and correspond to the projection of OTUs in the samples-space, so distance between samples reflects their specificity.

A majority of sequences (64%) obtained at the end of the incubation period belonged to 59 OTUs that were common to all the samples. However, 273 OTUs representing 10% of sequences were sample-specific. The phylogenetic profiles of the bacterial communities present in the bioreactor at the end of the incubation were dominated by Firmicutes (particularly of the Clostridia class) and contained lower levels of Proteobacteria- and Bacteroidetes-related OTUs (Figure 3). Moreover, unlike the original gut microbiome, the abundant OTUs present at the end of the experiment were the same for all samples, irrespective of the profile of the original inoculum (Table 3). PCoA analysis based on weighted-Unifrac distances confirmed that the final communities were significantly different compared to the initial gut microbiome (Figure 5). It is noteworthy that replicates of the gut microbiome of a given termite species clustered together, whereas replicates of the bioreactor communities were more distant, with the exception of those derived from *T. hospes*. Regarding the microbial communities present in the bioreactors, these can be separated into two subgroups, according to differences at the phylum level. The first group was dominated by Firmicutes (over 70%) and characterized by the absence of Bacteroidetes, while the second group was mainly composed of Bacteroidetes (20 to 50%) and Firmicutes (30 to 47%).

**Table 3:** Relative abundance (%) of the main phyla present in termite gut microbiomes and in the final bioreactor communities (colored lines).

**Species**	***M. parvus***				***N. ephratae***				***N. lujae***				***T. hospes***			
	**gut1**	**gut2**	**r1**	**r2**	**gut1**	**gut2**	**r1**	**r2**	**gut1**	**gut2**	**r1**	**r2**	**gut1**	**gut2**	**r1**	**r2**
**Bacteroidetes**	4.4	4.6	0.04	42.7	5.9	8.2	0.3	19.7	3.0	5.7	38.2	49.2	14.1	16.0	0.1	0.5
*Dysgonomonas* Otu002	–	–	–	98.4	–	–	22.2	–	0.7	2.6	99.3	53.4	2.6	–	–	–
*Dysgonomonas* Otu015	–	–	–	0.02	–	–	–	–	–	–	–	46.1	–	–	–	–
*Bacteroides* Otu018	–	0.1	–	–	–	–	–	93.8	–	–	–	–	–	–	–	–
**Firmicutes**	1.5	1.4	83.6	40.0	6.2	6.3	85.8	39.6	3.2	2.9	47.4	30.4	45.3	46.8	75.4	72.0
*Clostridium termitidis* Otu001	–	–	27.2	60.9	–	–	33.1	34.8	0.2	–	5.8	50.6	0.1	–	17.6	10.7
uncl *Lachnospiraceae* Otu003	0.4	0.9	8.2	–	0.3	0.7	14.9	2.6	0.6	2.3	44.3	–	0.7	–	45.2	32.9
uncl *Lachnospiraceae* Otu004	0.9	–	9.2	30.4	–	0.6	21.1	34.5	0.2	–	9.7	37.5	0.1	–	11.2	27.7
*Acetanaerobacterium* Otu014	–	–	26.8	–	–	–	1.0	–	0.8	–	–	–	–	–	0.3	0.02
*Ruminococcaceae* Gut_cluster Otu019	–	–	0.1	–	–	–	0.2	–	0.4	–	14.0	–	0.2	–	12.8	0.1
*Ruminococcaceae* Gut_cluster_7 Otu023	–	–	3.4	–	0.1	–	8.5	–	–	–	3.5	–	0.03	–	–	–
*Ruminococcaceae* Gut_cluster Otu024	–	–	1.6	1.1	–	–	2.2	7.3	–	–	5.9	0.5	0.04	–	0.4	2.2
*Sedimentibacter* Otu027	–	–	1.4	0.1	–	–	4.0	2.8	–	–	0.0	–	–	–	3.6	3.0
uncl *Clostridia* Otu034	–	–	9.1	–	–	–	–	–	–	–	–	–	–	–	–	–
*Lachnospiraceae Incertae_Sedis* Otu037	–	–	–	–	–	–	–	10.8	–	–	0.03	–	–	–	1.8	2.1
uncl *Clostridia* Otu042	–	–	3.0	–	–	–	4.2	–	–	–	–	–	–	–	–	–
uncl *Lachnospiraceae* Otu043	–	–	4.0	0.7	–	–	1.4	0.7	0.2	–	0.2	0.8	–	–	0.03	0.7
*Ruminococcaceae* Gut_cluster Otu053	–	–	–	–	–	–	–	–	–	–	3.7	–	0.04	–	0.4	3.0
**Proteobacteria**	1.9	2.3	15.8	17.3	1.6	2.2	13.5	40.5	3.5	2.7	14.2	20.4	11.9	10.8	24.4	27.5
uncl *Enterobacteriaceae* Otu005	–	–	0.1	38.2	0.4	1.2	21.7	81.2	0.2	0.5	43.1	55.6	0.4	0.7	8.0	14.2
*Pseudomonas_2* Otu010	–	–	0.6	32.1	0.4	–	4.8	2.5	0.4	–	21.5	16.5	0.2	–	63.5	33.3
uncl *Enterobacteriaceae* Otu012	–	–	0.0	12.9	–	–	3.7	1.0	–	–	26.2	22.1	0.2	–	1.4	47.3
*Stenotrophomonas* Otu028	–	–	44.7	0.3	–	–	24.5	–	–	–	1.0	–	–	–	–	–
uncl *Rhodocyclales* Otu029	–	–	10.1	–	0.4	–	25.0	–	0.2	–	6.4	–	0.2	–	15.9	–
*Escherichia*–*Shigella* Otu031	0.7	5.6	37.1	–	0.9	1.2	17.6	0.1	0.6	8.6	0.1	–	0.3	–	0.2	0.5
*Acinetobacter* Otu040	–	–	–	2.9	–	–	–	12.9	–	–	–	0.03	–	–	–	2.0

*For each phylum, OTU composition is detailed as relative abundance (%)*.

The phylogenetic distribution of 16S rRNA phylotypes was strikingly different in the bioreactors compared to those in the gut inocula. Notably, sequences related to Spirochaetes, Fibrobacteres and TG3, had almost disappeared at the end of the fermentation period (Figure 3), whereas sequences related to Bacteroidetes were highly abundant in bioreactors inoculated with gut microbiome from *N. lujae*, as well as in one of the bioreactors inoculated with *M. parvus* and *N. ephratae* gut microbiome. This phylum was represented by two OTUs belonging to the genera *Dysgonomas* and *Bacteroides*. Furthermore, within the bioreactor-derived communities, Proteobacteria-related OTUs belonging to the gamma*-*Proteobacteria class were identified, with some members being related to the *Pseudomonas, Escherichia, Acinetobacter* genera and another to an unclassified genus. Regarding the Firmicutes-related OTUs, these were composed of the class Clostridia, mainly related to Lachnospiraceae and Ruminococcaceae orders. *Clostridium termitidis* was found at high frequency in all reactor samples, representing on average 16% of the sequences, whereas sequences assigned to this species represented <0.02% of reads in the gut inocula.

The reactor-mediated enrichment of Firmicutes, Proteobacteria, and Bacteroidetes occurred at the expense of the three dominant phyla observed in the termite guts. Bacteria that were able to grow in the conditions prevailing in the bioreactors represented <5% in the initial gut community, and in some cases they represented <0.01% (Table 3).

## Discussion

The goal of this study was to determine the ability of termite gut microflora to grow in controlled bioreactor conditions, using raw wheat straw as the sole carbon source to produce carboxylates. Accordingly, our results demonstrate that gut microbiome from *N. ephratae, N. lujae, M. parvus*, and *T. hospes* were all able to degrade wheat straw and produce the targeted products. The highest level of wheat straw conversion was obtained in bioreactors inoculated with the *N. ephratae* gut microbiome, which was accompanied by high xylanase and cellulase activities.

Importantly, the extent of wheat straw degradation brought about by the termite gut microbiome was high compared to similar experiments performed using larger amounts of cow rumen inoculum (Lazuka et al., 2015). This is particularly true in the case of the *Nasutitermes lujae* gut inoculum (40 ± 0.5% wheat straw degradation), even if carboxylate production was variable in replicate bioreactors. Regarding this variability, it is important to note that the different termite individuals were collected from their nests over a 3-month interval (Material and Methods section) and, in the specific case of *N. lujae*, the inoculum size of the two replicate experiments was different, with a two-fold difference in the number of 16S rRNA gene copies (Table 1). Nevertheless, variable carboxylate production was also observed for the *M. parvus*-related inocula, despite a similar 16S rRNA gene copy count (i.e., a 15% difference) and in both cases a rather consistent bacterial community composition was observed. Therefore, it appears that other factors, such as the specific physiological state of the gut microbiome at the time of the gut's withdrawal, could be responsible for experimental variability.

In an attempt to relate wheat straw degradation to enzyme activity, cellulose, and xylanase activities were monitored. Significantly, the majority of these activities were cell-bound, which might be considered counter intuitive, since the enzymes must be acting on large insoluble polymers. However, the term cell-bound covers all cell-associated enzyme activities, including those present in the cyctoplasm, in the periplasmic space (Gram negative species) and those bound to the outer cell wall (e.g., enzymes associated with cellulosomes). In this respect, it is noteworthy that the bacterial communities in the bioreactors contained Clostridia, a class that contains cellulosome-producing members (He et al., 2013). Furthermore, while the reactor communities derived from the guts of *N. ephratae, N. lujae* and *M. parvus* all produced similar levels of cell-bound cellulase and xylanase activities, those from *N. ephratae* and *N. lujae* displayed the highest extracellular enzyme activities, and in particular more CMCase activity, which might explain why these communities also produced the highest level of wheat straw degradation. In contrast, bioreactors inoculated with gut microbiome from *T. hospes* displayed the lowest xylanase and cellulase activities. This observation is consistent with the observed low wheat straw degradation. In a previous study involving the use of switchgrass and corn stover as carbon sources for the growth of a compost inoculum, the production of 58 UA xylanase and 8 UA cellulase was correlated with approximately 34% w/w switchgrass degradation, while 23% corn stover degradation was achieved in the presence of <4 UA xylanase and 1 UA cellulase (Reddy et al., 2013).

Enzyme-mediated biomass deconstruction is a complex process involving whole arsenals of enzymes representing different families, substrate specificities and chemical mechanisms. Moreover, it involves intricate enzyme interplay and significant synergistic effects (Kumar et al., 2008; Wei et al., 2009). Therefore, it is to be expected that wheat straw degradation varies according to the exact enzyme mixture present in the bioreactor. Accordingly, further identification and characterization of the enzymes present in the different reactors should provide insight into the relationship between the composition of the enzyme arsenals produced by the different termite gut-derived inocula and their wheat straw-degrading capabilities.

A second aim of this study was to characterize the different components of the termite gut microbial communities and identify those that are involved in wheat straw degradation in the bioreactor experiments. The analysis of sequencing data showed that termite-gut microbial diversity decrease after incubation on wheat straw bioreactors irrespective the termite species origin of guts used as inocula. In this study, bioreactors were operated in batch mode which implies that bacteria displaying the highest growth rates under defined conditions are selected. Thus, diversity decrease reflects the selection of more adapted species growing faster on the lignocellulosic substrate under the experimental conditions applied in bioreactors. This in turn would result on the selection of the most efficient lignocellulolytic microorganisms. These observations are consistent with previous studies reporting a decline of diversity on microbial communities enriched on lignocellulosic substrates, decline that was particularly strong at the initial steps of the enrichment process (Reddy et al., 2011; Lazuka et al., 2015).

Additionally, among the termite species studied, *M. parvus, N. ephratae*, and *N. lujae* gut microbiome were dominated by members belonging to Spirochaetes and abundant in the Fibrobacteres phylum, though this was not the case for *T. hospes*, which was rich in Firmicutes-related OTUs. In the light of termite phylogenetic relationships, established by comparing the genes encoding cytochrome oxidase subunit II, this is surprising because *Nasutitermes* genus is more closely related to the *Termes* genus than to *Microcerotermes* (Supplementary Data S2). However, it is notable that *T. hospes* is the only humus-feeding species in this study, the three others being wood-feeders. Therefore, these results appear to confirm that termite gut microbiomes are mainly shaped by the host's diet, consistent with previous results (Mikaelyan et al., 2015a). Additionally, it is noteworthy that our data describing the *T. hospes* gut microbial community were similar to those reported for *T. comis* (Thongaram et al., 2005), indicating that the bacterial profiles are robust within the *Termes* genus. Similarly, the gut microbiome phylogenetic profiles of the wood-feeders *N. ephratae* and *M. parvus* closely resembled that of the *N. takasagoensis* microbiome (Hongoh et al., 2006), once again supporting the idea that gut microbiome profiles are shaped by feeding regimes. The weighted-Unifrac distance of the gut microbial communities from *M. parvus, N. lujae*, and *N. ephrataeae* (all three being wood-feeders) were highly similar (Figure 4). This signifies that these termite gut communities share numerous OTUs, but their relative abundances might vary between these species. Indeed, the weighted-Unifrac distance observed between *N. ephratae* and *N. lujae* gut communities results from differences in abundance of Fibrobacteres compared to the two other termite gut communities. The presence of TG3-related OTUs in all termite guts confirmed that this hypothetically new phylum is widespread and dominant in termite gut communities. TG3-related OTUs were surprisingly well grouped by host (Supplementary Data S3), supporting the hypothesis of co-evolution and host-adaptation processes proposed by Hongoh et al. (2005). It has been reported that TG3 members associate with fibers in the termite hindgut, leading to the postulate that these are lignocellulolytic bacteria (Mikaelyan et al., 2014). The two other major taxa observed in the termite gut samples were Spirochaetes and Fibrobacteres, which are not well characterized. Nevertheless, they have previously been described in ruminants as cellulose degraders (Kobayashi et al., 2008) and in higher termites as fiber-associated lignocellulose degraders (Warnecke et al., 2007; Mikaelyan et al., 2014). *Spirochaetaceae* have been shown to play a vital role in termites (Eutick et al., 1978), producing acetate from H_2_ and CO_2_ (Brauman et al., 1992), an interesting metabolic route for carboxylate production. Nevertheless, despite the high initial abundance of Spirochaetes and Fibrobacteres, neither of these phyla were maintained in the bioreactors, being absent at the end of the 20-day incubation period.

After incubation on wheat straw, comparison of microbial diversity between the initial and final states (based on Shannon's and Simpson's reciprocal indices) revealed that diversity was strongly decreased. For all termite gut inocula, the conditions prevailing in the bioreactors led to the selection of OTUs mainly related to Firmicutes, Bacteroidetes and Proteobacteria. This was particularly noticeable in the bioreactors inoculated with the *N. lujae* gut microbiome, which generated very reproducible community profiles, mainly composed of these three phyla. Regarding Proteobacteria, members of this phylum included *Enterobacteriaceae, Pseudomonas, Acinetobacter, Stenotrophomonas* and *Rhodocyclales*. These groups are not known for their ability to degrade lignocellulose, but they are reputed to ferment carbohydrates derived from lignocellulose degradation (Imhoff, 2005). Moreover, it is noteworthy that *Stenotrophomonas, Pseudomonas* and *Acinetobacter* normally display aerobic metabolism. Their presence in the bioreactors is thus surprising since experiments were conducted under strict anaerobic conditions and dissolved oxygen was never detected in the liquid phase (data not shown). Nevertheless, their relative abundance in the bioreactors was low, with maximal abundances of 3.6% *Stenotrophomonas* and 2.7% *Acinetobacter* in bioreactors inoculated with guts from *M. parvus and N. ephratae*, respectively. Higher abundance values were observed for *Pseudomonas* (12.3% in bioreactors inoculated with *T. hospes* guts), but facultative anaerobic metabolism has been reported for *Pseudomonas*, particularly, in relation to aromatics and lignoaromatics metabolism (Taylor, 1983; Liang et al., 2014). In this respect, it is notable that strict anaerobes such as *Clostridium, Bacteroides* or *Acetanaerobacterium* were also observed in the bioreactor communities. This apparent anomaly remains to be explained. In all the bioreactors, OTUs belonging to *Clostridia* (Firmicutes), particularly *Clostridium termitidis* OTU_1_, but also OTUs related to *Lachnospiraceae* (OTU_3, 4, 37, 43_) and *Ruminococcaceae* (OTU_19, 23, 24, 53_) were strongly enriched. *C. termitidis* was present in all bioreactors at an average of 16% of total reads. This species, previously identified in the *N. lujae* gut (Hethener et al., 1992), is reputedly a cellulose degrader able to use various sugars, including xylose as a source of carbon. So far, the role played by *Ruminococcaceae* and *Lachnospiraceae* in lignocellulose degradation has only been studied in mammals (Biddle et al., 2013), although previous studies have revealed their presence in termite environments, mainly in association with fungus-growing or humus and soil feeder termites (Mikaelyan et al., 2015a). Additionally, it has been shown that members of *Ruminococcaceae* and *Lachnospiraceae* are characterized by a greater number of plant cell wall-degrading glycoside hydrolase (GH) genes than those of the Clostridiaceae family. This is remarkable, because many species belonging to the Clostridiaceae family are lignocellulolytic, being characterized by the fact that they bear cellulosomes and their genomes encode large numbers of xylanases (He et al., 2013). Nevertheless, our observations suggest that the members of the Firmicutes phylum, which were enriched in all bioreactors, are the main source of wheat straw degradation in the experimental conditions employed in this study. OTUs related to Bacteroidetes were also enriched in some of the reactors, particularly *Dysgonomonas* (OTU_2, 15_) and *Bacteroides* (OTU_18_). Members of the *Dysgonomonas* genus are known for their lignocellulolytic potential (Sun et al., 2015) and have been identified as putative cellulose degraders in termite gut (Yang et al., 2014). Species in this genus are also able to degrade cellobiose and glucose (Hofstad et al., 2000). *Bacteroides* species are commonly found in mammalian digestive tracts and have been described as xylanolytic (Nishiyama et al., 2009).

The final composition of the bacterial communities growing on wheat straw in the bioreactors was clearly distinct when compared to the parental termite gut communities (Figure 5). Diversity analysis showed that OTUs present in termite guts were mainly host-specific. However, in the bioreactor experiments a certain convergence of OTUs was observed. This is no doubt caused by the identical conditions prevailing in the different bioreactors. Moreover, the community structure in the bioreactors more closely resembled lignocellulolytic communities present in rumen or soil than those in termite guts (He et al., 2013). While it is difficult to ascribe these changes to one particular factor, unsatisfied pH, nutrient and/or O_2_ requirements, altered host specific signaling and inter- taxa dependency relationships might explain the loss of certain phyla during our experiments.

It is difficult to compare the biomass-degrading potency of the termite gut microbiomes studied herein with those described in other studies, because the latter are often enriched microbial communities from compost, forest soils, or mangrove sediments grown on different substrates that were prepared in different ways (Feng et al., 2011; Reddy et al., 2011; Yan et al., 2012). For example, Feng et al. (2011) reported that a woodland soil inoculum growing at 40°C achieved 51% degradation of corn stover powder (mesh 40 or 375 μm) and 44% degradation of steam-exploded corn. In another study, a cow manure inoculum growing on alkali-pretreated rice straw in an anaerobic digester yielded 49% degradation of the substrate after 7-day incubation (Yan et al., 2012). However, to our knowledge, no previous investigations have focused on lignocellulose degradation by termite gut communities growing in bioreactor conditions. Nevertheless, it is possible to simply observe that wheat straw degradation levels in this study, particularly those obtained with the *Nasutitermes* gut microbiome (up to 45% w/w) are comparable with those mentioned above. Moreover, it is noteworthy that this level of degradation was achieved using a relatively intact substrate (simply milled to 2 mm) and in the absence of prior enrichment of microbial community. This suggests that it might be possible to achieve higher degradation levels using a pretreated substrate (Yan et al., 2012; Lazuka et al., 2017) or an enrichment strategy (Reddy et al., 2012; Lazuka et al., 2015). In the conditions applied here, a slightly acid pH was chosen in order to inhibit methanogenesis and favor carboxylate accumulation. However, the pH conditions prevailing in termite guts is variable and can reach very high pH values in the hindgut (P3) region, which harbors a dense microbial community and is the place where most lignocellulose degradation occurs. This is the case for some soil-feeding higher termites, such as those from the genus *Cubitermes* (Brune and Kühl, 1996), but it is untrue for termites belonging to the *Nasutitermes* genus. Their hindguts are characterized by neutral or even slightly acidic pH values (Köhler et al., 2012), close to the pH prevailing in the bioreactors used in this study. Indeed, this observation provides a possible explanation as to why the *Nasutitermes* microbiome performed best in our experimental conditions.

In conclusion, this study demonstrated that termite gut microflora can be grown in bioreactors, using lignocellulose as the sole carbon source to produce carboxylates. Further studies should focus on deeper characterization of the enriched microbial communities and identification of the key microbial and enzymatic drivers for lignocellulose bioconversion. Moreover, it will be worthwhile to investigate how modified experimental conditions can be used to further optimize the bioconversion process in order to maximize carboxylate production.

## Author contributions

LA, student on this project, did all bioreactors' experiments and data interpretation. He carried out the molecular diversity analysis and drafted the manuscript. AL, student in this project did the metabolite and enzymatic activity assays; she also trained LA to bioreactor utilization and monitoring. DS-D participated on termite rearing, selection and dissection; he trained LA to termite dissection. EM participated on termite selection for this project. DS-D and EM revised the manuscript critically. MO and GH-R participate on experimental design and project conception; they revised the manuscript critically for important intellectual content. GH-R is the main supervisor and MO is co-supervisor of LA and AL. GH-R is the project coordinator, conceived the study, and participated in its design and helped to draft the manuscript. All authors read and approved the final manuscript.

### Conflict of interest statement

The authors declare that the research was conducted in the absence of any commercial or financial relationships that could be construed as a potential conflict of interest.
