# Supplementary material for: Uncovering the Potential of Termite Gut Microbiome for Lignocellulose Bioconversion in Anaerobic Batch Bioreactors
Source: Front Microbiol. 2017 Dec 22;8:2623. doi: 10.3389/fmicb.2017.02623 (PMC5744482; doi:10.3389/fmicb.2017.02623)
Supplement: Supplementary file 1 [file DataSheet1.DOCX]

**Supplementary data**

**S1.** Rarefaction curves of the sequenced points.

Rarefaction curves were generated with the Mothur subroutine rarefaction.single on the randomly subsampled 15 k final sequences. For all the samples but *N. lujae* r2, the number of detected OTUs reached a plateau corresponding to its observed richness, indicating that sequencing depth is sufficient to describe the community (*M. parvus=*MP*, N. ephratae=*NE*,* N. *lujae=*NL*, T. hospes=*TH).

**S2.** Phylogenetic tree of representative termite species.

Sequences corresponding to the cytochrome oxidase subunit II gene were collected from NCBI. They were aligned using Clustal Omega and used to build a neighbor-joining tree between the four termite species of this study and two species, *Termes comis* and *Nasutitermes takasagoensis,* whose microbiome were described in other studies and were very close to respectively our *T. hospes* and *N. ephratae*.


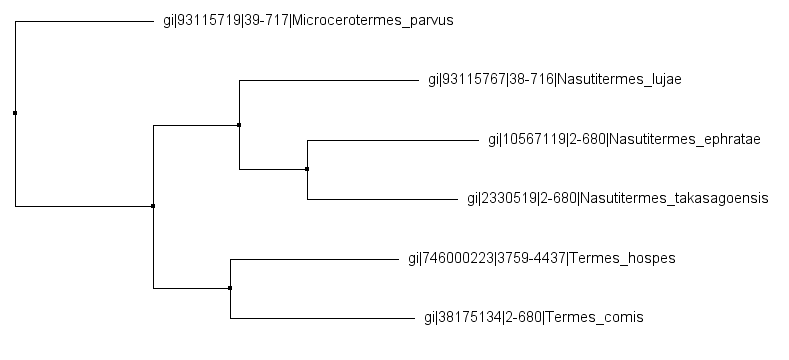


**S3.** Phylogenetic position of the TG3 OTUs


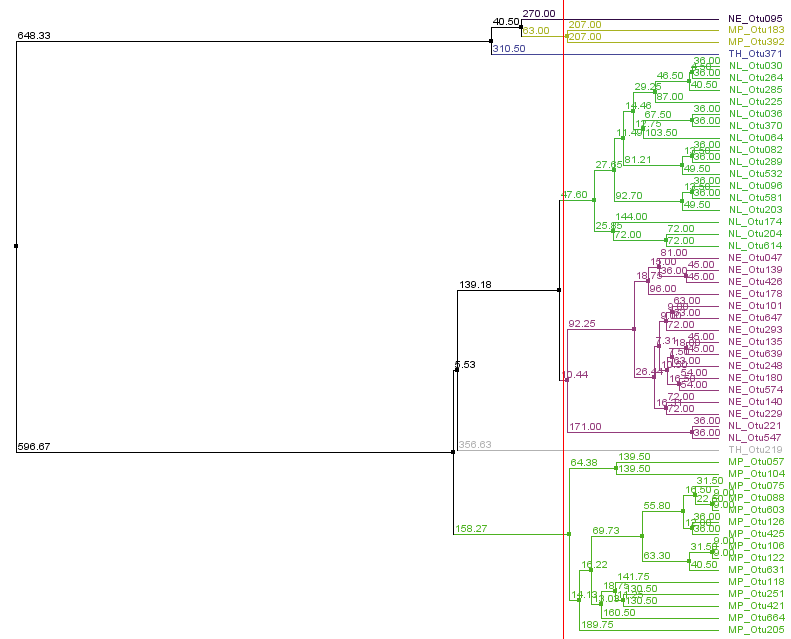


**Table S1.** Relative abundance (%) of main phyla present in termite gut microbiomes. For each phylum, major OTU composition (≥2% in at least one sample) is detailed.

|  | ***M. parvus*** | | ***N. ephratae*** | | ***N. lujae*** | | ***T. hospes*** | |
| --- | --- | --- | --- | --- | --- | --- | --- | --- |
|  | gut1 | gut2 | gut1 | gut2 | gut1 | gut2 | gut1 | gut2 |
| ***Bacteroidetes*** | 4.4 | 4.6 | 5.9 | 8.2 | 3.0 | 5.7 | 14.1 | 16.0 |
| *Dysgonomonas Otu002* | - | - | - | - | 0.7 | 2.6 | 2.6 | - |
| ***Firmicutes*** | 1.5 | 1.4 | 6.2 | 6.3 | 3.2 | 2.9 | 45.3 | 46.8 |
| *uncl Lachnospiraceae Otu003* | 0.4 | 0.9 | 0.3 | 0.7 | 0.6 | 2.3 | 0.7 | - |
| *Lactococcus Otu020* | - | - | - | - | - | - | 15.2 | 14.5 |
| *Ruminococcaceae insect cluster Otu026* | - | - | - | - | - | - | 12.3 | 11.7 |
| *uncl Ruminococcaceae Otu045* | - | - | - | - | - | - | 6.1 | 6.0 |
| *uncl Ruminococcaceae Otu046* | - | - | - | - | - | - | 5.6 | 5.6 |
| *Uncultured_24 Ruminococcaceae Otu048* | 2.2 | 1.4 | 0.1 | - | - | - | 4.6 | 6.2 |
| *Turicibacter sanguinis Otu051* | - | - | - | - | - | - | 5.2 | 4.4 |
| ***Proteobacteria*** | 1.9 | 2.3 | 1.6 | 2.2 | 3.5 | 2.7 | 11.9 | 10.8 |
| *Escherichia-Shigella Otu031* | 0.7 | 5.6 | 0.9 | 1.2 | 0.6 | 8.6 | 0.3 | - |
| *uncl Myxococcales Otu041* | - | - | - | - | - | - | 27.4 | 26.7 |
| ***Fibrobacteres*** | 15.2 | 7.4 | 20.3 | 8.8 | 1.7 | 0.7 | 0.5 | 0.4 |
| *Fibrobacteres termite subcluster Ib Otu017* | - | - | 66.1 | 59.9 | - | - | - | - |
| *Fibrobacteres termite subcluster Ia Otu032* | 34.3 | 34.5 | - | - | - | - | - | - |
| *Fibrobacteres termite subcluster Ia Otu033* | 34.3 | 34.5 | - | - | - | - | - | - |
| *Fibrobacteres termite cluster_I Otu049* | - | - | 15.7 | 20.6 | - | - | - | - |
| *Fibrobacteres termite subcluster Ib Otu052* | - | - | 14.6 | 15.0 | - | - | - | - |
| *Fibrobacteres termite subcluster_Ia Otu070* | 14.1 | 13.3 | - | - | - | - | - | - |
| ***Spirochaetes*** | 62.8 | 72.7 | 54.2 | 65.0 | 70.2 | 72.9 | 21.6 | 20.0 |
| *Treponema_If Otu006* | - | - | 35.3 | 33.1 | 9.5 | 8.4 | - | - |
| *Treponema_Ic Otu007* | - | - | 9.5 | 11.4 | 26.7 | 26.6 | - | - |
| *Treponema_Ic Otu008* | 33.6 | 34.8 | - | - | - | - | - | - |
| *Treponema_Ic Otu009* | - | - | 12.9 | 14.0 | 20.6 | 19.3 | - | - |
| *Treponema_If Otu011* | - | - | 13.9 | 10.8 | 8.6 | 7.3 | - | - |
| *Treponema_If Otu013* | 19.9 | 16.1 | - | - | - | - | - | - |
| *Treponema_Ic Otu016* | 16.4 | 16.3 | - | - | - | - | - | - |
| *Treponema_Ia Otu021* | - | - | 2.7 | 3.7 | 5.9 | 8.9 | 0.03 | - |
| *Treponema_Ia Otu022* | - | - | 10.1 | 12.0 | - | - | - | - |
| *Treponema_If Otu025* | - | - | 1.9 | 1.3 | 7.5 | 5.8 | 0.03 | - |
| *uncl Treponema_I Otu035* | - | - | 0.05 | - | 4.9 | 5.6 | - | - |
| *uncl Treponema_I Otu038* | 0.2 | 0.4 | 0.1 | - | 4.2 | 4.9 | - | - |
| *Treponema_Ia Otu039* | 4.6 | 4.9 | - | - | - | - | - | - |
| *Treponema_Ia Otu044* | 3.5 | 5.0 | - | - | - | - | - | - |
| *Treponema_Ic Otu050* | - | - | - | - | 3.3 | 3.5 | - | - |
| *uncl Treponema_I Otu059* | - | - | - | - | - | - | 9.6 | 7.7 |
| *Treponema_Ic Otu062* | - | - | 2.7 | 3.2 | - | - | - | - |
| ***TG3*** | 11.2 | 8.7 | 9.3 | 7.7 | 16.6 | 14.0 | 0.5 | 0.5 |
| *TG3 subcluster IIIa Otu030* | - | - | 0.4 | - | 29.6 | 29.8 | - | - |
| *TG3 termite cluster III Otu036* | - | - | 0.1 | - | 26.2 | 20.4 | - | - |
| *TG3 subcluster_IIIa Otu047* | - | - | 30.5 | 30.0 | - | - | - | - |
| ***Others*** | 2.9 | 2.9 | 2.6 | 1.9 | 1.7 | 1.1 | 6.2 | 5.5 |
